# Long COVID: Distinction between Organ Damage and Deconditioning

**DOI:** 10.3390/jcm10173782

**Published:** 2021-08-24

**Authors:** Johannes Kersten, Michael Baumhardt, Paul Hartveg, Luis Hoyo, Elina Hüll, Armin Imhof, Cornelia Kropf-Sanchen, Nicoleta Nita, Johannes Mörike, Manuel Rattka, Stefanie Andreß, Dominik Scharnbeck, Gerlinde Schmidtke-Schrezenmeier, Marijana Tadic, Alexander Wolf, Wolfgang Rottbauer, Dominik Buckert

**Affiliations:** Department for Internal Medicine II, University of Ulm, 89081 Ulm, Germany; michael.baumhardt@uniklinik-ulm.de (M.B.); paul.hartveg@uniklinik-ulm.de (P.H.); luis.hoyogarcia@uniklinik-ulm.de (L.H.); elina.huell@uni-ulm.de (E.H.); armin.imhof@uniklinik-ulm.de (A.I.); cornelia.kropf-sanchen@uniklinik-ulm.de (C.K.-S.); nicoleta.nita@uniklinik-ulm.de (N.N.); johannes.moerike@uniklinik-ulm.de (J.M.); manuel.rattka@uniklinik-ulm.de (M.R.); stefanie.andress@uniklinik-ulm.de (S.A.); dominik.scharnbeck@uniklinik-ulm.de (D.S.); gerlinde.schmidtke-schrezenmeier@uniklinik-ulm.de (G.S.-S.); marijana.tadic@uniklinik-ulm.de (M.T.); alexander.wolf@uniklinik-ulm.de (A.W.); wolfgang.rottbauer@uniklinik-ulm.de (W.R.); dominik.buckert@uniklinik-ulm.de (D.B.)

**Keywords:** COVID-19, long COVID, Post-COVID, cardiac sequelae, pulmonary sequelae

## Abstract

(1) Background: Long COVID syndrome refers to long-term sequelae of the novel viral disease, which occur even in patients with initially mild disease courses. However, there is still little evidence of the actual organic consequences and their frequency, and there is no standardized workup to diagnose long COVID syndrome yet. In this study, we aim to determine the efficiency of a stepwise diagnostic approach for reconvalescent COVID-19 patients with cardiopulmonary symptoms. (2) Methods: The diagnostic workup for long COVID syndrome included three steps. In the first step, the focus was on broad applicability (e.g., blood tests and body plethysmography). In the second step, cardiopulmonary exercise testing (CPET) and cardiac MRI (CMR) were used. The third step was tailored to the individual needs of each patient. The observation period lasted from 22 February to 14 May 2021. (3) Results: We examined 231 patients in our long COVID unit (mean [SD] age, 47.8 [14.9], 132 [57.1%] women). Acute illness occurred a mean (SD) of 121 (77) days previously. Suspicious findings in the first visit were seen in 80 (34.6%) patients, prompting further diagnostics. Thirty-six patients were further examined with CPET and CMR. Of those, 16 (44.4%) had pathological findings. The rest had functional complaints without organ damage (“functional long COVID”). Cardiopulmonary sequelae were found in asymptomatic as well as severe courses of the initial COVID-19 disease. (4) Conclusions: A structured diagnostic pathway for the diagnosis of long COVID syndrome is practicable and rational in terms of resource allocation. With this approach, manifest organ damage can be accurately and comprehensively diagnosed and distinguished from functional complaints.

## 1. Introduction

The ongoing COVID-19 pandemic is placing heavy burdens on health care systems, including requiring high intensive care unit (ICU) capacities to jeopardizing established processes of medical care [1,2]. While new confirmed cases and deaths are being added daily, the number of patients who have recovered is also steadily increasing. Severe effects on various organ systems in the context of acute disease are already known [3,4,5,6]. However, the number of patients with more diffuse symptoms, including neuropsychiatric and cardiopulmonary symptoms, is rising. Such cases are currently described by the term “long COVID syndrome.” Possible explanations include inflammation of single organs, residual debris in tissue, such as in the resorption stage after pneumonia, and a transiently changed metabolism. Little is currently known about the causes. What is clear, however, is the heavy symptom burden of those affected and the urgent need for specialized contact points.

Given the diversity of symptoms, the prevalence of long COVID remains unclear. Moreover, due to a lack of structured workups for this patient population, estimates currently rely on inferences from individual cases with a potential for coincidence in all COVID-19 reconvalescents. Developing a structured workup is of great importance, given the enormous number of people who have recovered from COVID-19 worldwide.

Here, we report our diagnostic workup for long COVID patients and share our first experiences. The approach is based on an escalation of various diagnostic modalities, with a focus on sensible resource allocation combined with a comprehensive workup. Although cardiopulmonary symptoms are the primary focus, an interdisciplinary approach is ultimately adopted when necessary.

## 2. Materials and Methods

This was a single-center, prospective, observational study conducted in a German tertiary care center. Since 22 February 2021, we have been operating a special outpatient clinic for patients with persistent symptoms after recovering from COVID-19. All patients had a previous positive polymerase chain reaction (PCR) test for SARS-CoV-2 and had been released from quarantine at least 1 month previously. A selection of previously hospitalized patients was deliberately not conducted since relevant post-infectious symptoms have been described even in mildly symptomatic COVID-19 courses [7]. All patients were referred to our center by their general practitioner because of persisting symptoms. This study was approved by the ethics committee of the University of Ulm (approval No. 406/20). All patients provided written informed consent.

**Figure 1:**
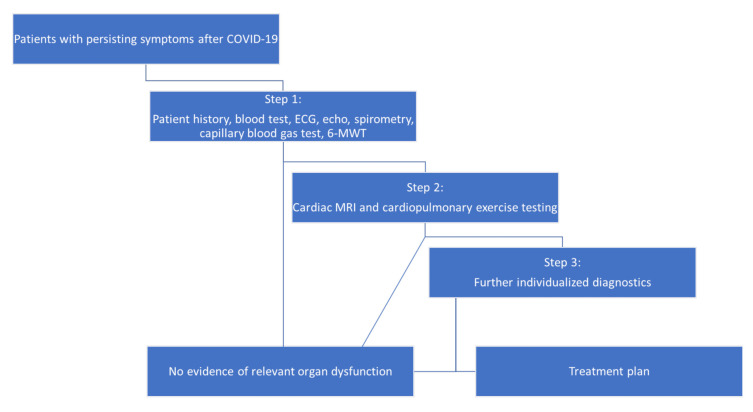
Stepwise diagnostic workup of patients with long COVID syndrome. ECG, electrocardiogram; MRI, magnetic resonance imaging; 6-MWT, 6-min walk test.

The diagnostic workup followed a strict stepwise protocol, as shown in Figure 1. In Step 1, laboratory tests, a 12-lead electrocardiogram, transthoracic echocardiography, body plethysmography (including diffusing capacity), a capillary blood gas test, and a 6-min walk test (6-MWT) were performed. The blood test consisted of standard parameters, such as electrolytes, renal parameters, coagulation values, transaminases, C-reactive protein, thyroid parameters, and a differential blood count. Additionally, values for troponin T and N-terminal pro b-type natriuretic peptide (NT-proBNP) were examined. Antibodies to the SARS-CoV-2 surface protein were determined using a commercially available electrochemiluminescence immunoassay kit (Roche). Transthoracic echocardiography was performed according to current guidelines, including left ventricular ejection fraction (LVEF) evaluation using the modified biplane Simpson method [8]. Left ventricular global longitudinal strain (LV GLS) was quantified using commercially available software (TOMTEC, Munich, Germany) using the three apical views.

Step 2 of the diagnostic workup consisted of cardiopulmonary exercise testing (CPET) and cardiovascular magnetic resonance imaging (CMR). The decision to proceed to Step 2 was made on the basis of all anamnestic and diagnostic data gathered in Step 1. Pathological values in Step 1 or an inacceptable symptom burden with a clear or at least possible occurrence in the context of COVID-19 led to further diagnostics. The patient could refuse further examinations at any time. Changes that could clearly be traced back to a preexisting illness and that had already been described before COVID-19 were not used as absolute criteria for the decision. CPET was performed and interpreted using a treadmill ramp protocol with continuous measurement of ventilatory gases and repeated capillary blood gas tests according to current recommendations [9,10]. CMR scans were obtained using a 1.5 T scanner (Achieva; Philips Medical Systems, Best, The Netherlands) under a standardized protocol in accordance with the guidelines of the Society for Cardiovascular Magnetic Resonance for the evaluation of inflammatory myocardial diseases [11].

In Step 3, further diagnostics were performed on an individual basis. Patients were referred to other medical disciplines for specific and potentially invasive diagnostics (e.g., pneumology for bronchoscopy, cardiology for right heart catheterization, neurology, or psychiatry).

The practicability of our stepwise approach was first assessed in a small cohort of 20 patients, which are included in this study.

### Statistics

Descriptive analysis results are reported as means (standard deviations; SDs) or numbers (percentages). Because the cohort, especially the fraction that completed the second diagnostic step, was not as large as anticipated at the time of performing this preliminary analysis, the analysis was performed on a descriptive rather than comparative basis. In line with this, no comparative statistical tests were used for comparisons between hospitalized and non-hospitalized patients. Patients with and without a clear indication for organ damage in the further workup were compared using Fisher’s exact test according to the small sample size, unless otherwise mentioned. Due to its exploratory nature and the lack of data at the beginning of the study, no sample size calculation or power analysis prior to the beginning of enrollment was applicable.

## 3. Results

From 22 February until 14 May 2021, a total of 231 patients referred to our long COVID unit for the diagnosis of potential post-inflammatory organ damage. The patient characteristics are summarized in Table 1. Most patients were women and had no relevant previous illnesses. Oligo- or asymptomatic and severe cases constituted a small proportion of the entire cohort. Acute illness had occurred a median (SD) of 122 (77) days prior to referral. Most patients complained of shortness of breath, chest pressure, fatigue, and memory or concentration problems.

**Figure 2:**
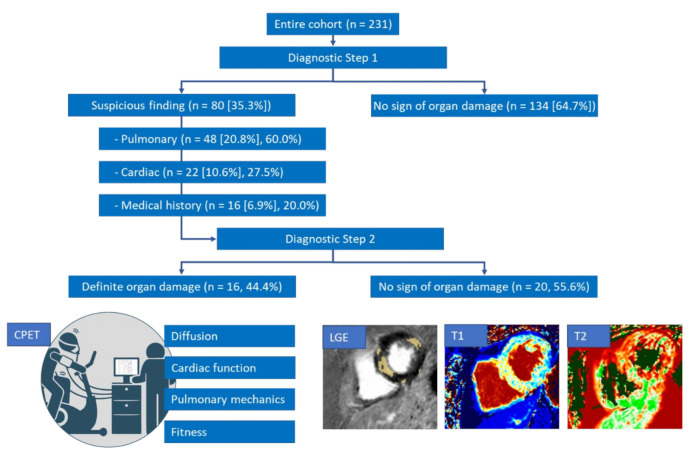
Diagnostic stepwise workup of 231 patients with long COVID. CPET, cardiopulmonary exercise testing; LGE, late gadolinium enhancement. The percentage in square brackets is according to the entire cohort, the other percentage refers to the proportion of patients in the distinct diagnostic step.

In Step 1 of the diagnostic workup, relevant long-term consequences of COVID-19 were excluded in 151 (65.4%) patients. No changes in any of the apparatus-based examinations or “red flags” in the anamnesis were found in these patients. For the remaining 80 (34.6%) patients, further diagnostics were arranged, mainly due to abnormal findings in pulmonary diagnostics (*n* = 48 [60%]; 20.8% of the entire cohort), including body plethysmography, capillary blood gas analysis, and 6-MWT. Minor diffusion disturbances (diffusing capacity of the lungs for carbon monoxide [DLCO] below 80%) and desaturation in the 6-MWT were decisive factors. Cardiac abnormalities were less common (*n* = 22; 10.7% of the entire cohort), with some overlap with tentatively pulmonary sequelae, mainly in terms of LVEF (< 55%) and LV GLS (> −15%). Details of the test results are shown in Table 2 In 16 (6.9%) cases; only suspicious anamnesis led to further diagnostics, especially subjective dyspnea causing ongoing incapacity to work or prolonged hospitalization. An overview of the actual diagnostic workup is shown in Figure 2.

Of the 80 patients with suspicious findings in Step 1 of the workup, 36 completed Step 2 (CPET and CMR). There were no cardiopulmonary limitations in 14 (45.2%) of the 31 patients with a fully completed CPET. Signs of deconditioning were noted in 4 (12.9%) patients. A cardiac capacity limitation was observed in only one patient (3.2%), pulmonary-mechanical limitations (e.g., restriction, obstruction, and inadequate hyperventilation) were noted in five patients (16.1%), and pulmonary-vascular limitations were observed in six patients (19.4%). Apart from the low number of real cardiac performance limitations in CPET, CMR showed (post-)inflammatory cardiac sequelae in 10 (27.8%) of the 36 patients, with signs of myocarditis (such as nonischemic late gadolinium enhancement or pathologic findings in T1 or T2 mapping) in 9 cases and an unclear reduction in the left ventricular function in 1 patient. Four of the six patients with pulmonary-vascular performance limitations in CPET had pathological findings on CMR. Moreover, 16 (44.4%) of the 36 patients who completed Step 2 showed at least 1 pathological change in CPET or on CMR. Risk factors for this clear indication of organ damage were a higher body mass index and arterial hypertension, as seen in Table 3. Other factors, such as gender, age, and previous cardiac, pulmonary, or malignant diseases, did not seem to have an influence on the occurrence of COVID-19-induced organ damage, although our data are not conclusive due to the small case number. The test for SARS-CoV-2 surface protein antibodies was negative in one patient with organic damage and one patient with only functional complaints.

With pathologic findings in 44.4% of the 36 Step 2 patients out of 34.6% with suspicious findings in Step 1, we estimated cardiopulmonary sequelae in the meaning of real organ damage, such as myocarditis, in about 15.4% of patients in the entire cohort. Only 18 (7.8%) patients had been hospitalized because of severe COVID-19. Of those, only 3 (18.8%) were among the 16 patients with pathologic findings in Step 2. Due to the low number of cases, a comparative analysis between hospitalized and non-hospitalized patients was not possible at this stage.

## 4. Discussion

The main findings of this study are as follows: (1) our proposed stepwise diagnostic approach is suitable for patients with long COVID syndrome; (2) suspicious findings requiring further investigation were noted in 34.6% of the patients in our cohort; and (3) with this stepwise approach, a high percentage of cardiopulmonary sequelae was found using CPET and CMR.

Various organ manifestations of long COVID syndrome have already been described. They range from endocrinological and gastroenterological diseases to interstitial lung diseases and myocarditis [12,13,14,15,16,17,18]. Neuropsychiatric complaints, such as persistent fatigue and anosmia, which are perceived as extremely wearing, have also been reported. In a matched cohort analysis of 27,589 inpatients and 46,857 outpatients, long COVID occurred at rates of 7% and 7.7%, respectively [12]. Since these data are based on the collection of new ICD-10 codes after having contracted COVID-19, there is a possibility of underdiagnosis. Moreover, there are syndromic conditions with a clear loss of performance, which are obscured when classified as ICD-10. A structured diagnostic workup of symptomatic COVID-19 reconvalescents, such as that employed in our long COVID unit, may reveal higher numbers. On the other hand, we were able to show that actual organ damage is present in a minority of patients with persistent complaints. In contrast to the “organic long COVID”, this “functional long COVID” will remain a diagnostic and therapeutic challenge.

In our study, the most common symptoms were dyspnea, fatigue, memory and concentration disorders, and thoracic pain or pressure. These results are consistent with prior studies [19,20]. The Step 1 diagnostics were selected by our research group because they were readily available and allowed the examination of many patients in a short time. Their practicability was first assessed in a small cohort (data not shown) and confirmed by the results reported herein. Two-dimensional transthoracic echocardiography, including speckle tracking strain analysis, combined with a 12-channel electrocardiogram and myocardial biomarkers appeared to be sufficiently sensitive in the detection of myocardial sequelae. Previous findings support this assumption in the context of myocarditis [18,21,22]. The same is true for body plethysmography, including the diffusion capacity for CO, which has already been described in long COVID syndrome [23,24]. The 6-MWT was selected as a diagnostic tool for Step 1 because, despite its simplicity, it has been validated for many cardiopulmonary diseases [25,26], and a correlation between the walking distance and the presumed oxygen uptake in CPET has been reported [27]. In the context of our investigation, influencing factors, such as joint problems or fatigue, must be considered, particularly with regard to the walking distance. Our Step 2 diagnostics can be considered the gold standard for cardiopulmonary functional diagnostics. The diagnostic and prognostic quality of CPET has been proven for any cardiopulmonary disease. CMR is the standard diagnostic tool not only for cardiac volumetry and function but also for inflammatory or fibrosing myocardial diseases [28,29,30]. Our diagnostic stepwise approach is disputable according to costly laboratory tests, such as NT-proBNP, C-reactive protein, and antibody levels, without showing high positivity rates so far. The diagnostic accuracy of transthoracic echocardiography or body plethysmography according to the pretest probability in the presence or absence of typical cardiac or pulmonary symptoms should also be subject of further investigations. Likewise, it must be critically questioned whether CMR and CPET should be performed for every patient with any suspect finding in Step 1. The local availability and financial aspects have to be taken into account. The sensitivity and specificity of our diagnostic approach could not be tested in the absence of a defined gold standard. 

In this investigation, we did not use only the results of apparatus-based diagnostics to establish the indication for Step 2. Complaints of severe symptoms with no corresponding objective signs were also considered. This has the advantage that severely suffering patients are taken seriously. At the same time, our diagnostic approach offers the possibility of reliable exclusion diagnostics. A seven-fold increase in depression attributed to quarantines, lockdowns, and social distancing amid the COVID-19 pandemic has been reported [31]. Since depression and long COVID syndrome overlap with some neuropsychiatric symptoms, such as fatigue and concentration and sleep disorders, the exclusion of cardiopulmonary sequelae is crucially important for these patients. Our cardiopulmonary workup enables referral to other specialist disciplines, such as psychiatry or psychosomatics.

In our cohort, the number of severe courses of COVID-19 with hospitalization was relatively low. In other studies on cardiopulmonary sequelae, this group is often clearly overrepresented. In a study by Puntman et al., for example, there were cardiac abnormalities on CMR in 60 of the 100 patients examined, and 33% of the patients required hospitalization due to severe COVID-19 [16]. The authors found no significant differences in cardiac sequelae between hospitalized and non-hospitalized patients. In our study, there were some indications of such differences, especially from the results of Step 1 of the diagnostic workup. A larger cohort, for which we aim, should provide more evidence. In severe cases, factors other than SARS-CoV-2, such as post-ICU syndrome or bacterial superinfections, must also be considered causally. In light of this, our high number of reduced diffusion capacities in the group of hospitalized patients after, for example, invasive ventilation, does not seem surprising and is in line with studies showing a reduced diffusion capacity after severe COVID-19 [23,24]. It must also be noted that changes in the DLCO or functional capacity also occur after other viral or bacterial diseases with severe courses or after intensive medical care (post-ICU syndrome) [32,33,34,35,36,37]. It is, therefore, important to differentiate actual COVID-19 sequelae from the usual side effects of infectious diseases before defining a new entity. Perhaps classic healthy comparison collectives may not be useful for this purpose. We hope that our stepwise workup will help to manage long COVID symptoms.

## 5. Limitations

Selection bias may be inherent in this study, as it is conceivable that patients with more severe symptoms are more likely to refer to our unit. Therefore, conclusions regarding the actual prevalence of the various symptom complexes cannot be drawn. Moreover, an influence of press coverage on patient self-perceptions cannot be excluded. Furthermore, our cohort was relatively small, and a suitable comparative cohort was not available. This limitation also applies to the inability to test the sensitivity of the Step 1 diagnostics in the context of long COVID syndrome. However, our investigation is ongoing, and we hope to be able to present more detailed data in the near future.

## 6. Conclusions

Cardiac and pulmonary sequelae after COVID-19 are a common finding. There is a discrepancy between functional complaints and the actual occurrence of organ damage in the extended diagnostics. Our proposed step-by-step approach consisting of a Step 1 with basic diagnostics (anamnesis, blood test, transthoracic echocardiography, body plethysmography, capillary blood gas analysis, and a 6-min walk test), a Step 2 with extended diagnostics (cardiac magnetic resonance and cardiopulmonary excuse testing), and an individual Step 3 is a first suggestion for a sensible allocation of resources in view of the high number of patients affected. A further understanding of long COVID is needed to understand whether it is a new entity or a form of known syndromes, such as post-ICU syndrome or post-infection syndrome.

## Figures and Tables

**Table 1:** Patient Characteristics (*n* = 231).

Characteristic	Value
Age, mean (SD), year	47.8 (14.9)
Women, *n* (%)	132 (57.1)
Body mass index, mean (SD), kg/m^2^	25.6 (4.6)
COVID-19 history	
Oligosymptomatic/asymptomatic course, *n* (%)	34 (14.7)
Hospitalization, *n* (%)	18 (7.8)
Invasive ventilation, *n* (%)	6 (2.6)
Therapy with corticosteroids, *n* (%)	13 (5.5)
Therapy with antibiotics, *n* (%)	13 (5.5)
Intervening vaccination, *n* (%)	19 (8.2)
Other histories	
Cardiac diseases, *n* (%)	12 (5.2)
Pulmonary diseases, *n* (%)	29 (12.6) [asthma bronchiale, 23 (10.0)]
Malignant diseases, *n* (%)	9 (3.9)
Cardiovascular risk profile	
Arterial hypertension, *n* (%)	48 (20.8)
Diabetes mellitus type I, *n* (%)	12 (5.2)
Diabetes mellitus type II, *n* (%)	2 (0.9)
Dyslipidemia, *n* (%)	127 (55.0)
Current/past smoking, *n* (%)	46 (19.9)
Long COVID symptoms	
Thoracic pain/pressure, *n* (%)	56 (24.2)
Dyspnea, *n* (%)	114 (49.4)
Fever, *n* (%)	5 (2.2)
Anosmia/ageusia, *n* (%)	29 (12.6)
Headaches, *n* (%)	19 (8.2)
Sleep disorders, *n* (%)	26 (11.3)
Exhaustion/fatigue, *n* (%)	122 (52.8)
Memory and concentration disorders, *n* (%)	64 (27.7)
Blood test	
Hemoglobin, mean (SD), g/dL [normal, 12.3–15.3]	14.4 (1.1)
Glomerular filtration rate, mean (SD), mL/min	90.8 (16.6)
C-reactive protein, mean (SD), mg/L [normal, < 5.0]	2.8 (8.0)
Thyroid-stimulating hormone, mean (SD), mU/L [normal, 0.400–3.770]	1.76 (1.51)
D-dimers, mean (SD), mg/L FEU [normal, < 0.50]	0.27 (0.20)
Troponin T, mean (SD), ng/L [normal, < 15.0]	4.8 (3.4)
NT-proBNP, mean (SD), pg/mL [normal, < 130.0]	74.0 (67.6)
Antibody against SARS-CoV-2 surface protein (228 patients *), positive *n* (%)/negative *n* (%)	207 (90.8)/21 (9.2)

Abbreviations: FEU, fibrinogen-equivalent units; NT-proBNP, N-terminal pro b-type natriuretic peptide. * Three samples could not be tested for SARS-CoV-2 antibodies due to a temporary problem in the laboratory.

**Table 2:** Decisive findings in Step 1 of the proposed diagnostic approach.

Parameter	Entire Cohort	Asymptomatic/Oligosymptomatic (*n* = 34)	Symptomatic, Not Hospitalized	Hospitalized
	(*n* = 231)		(*n* = 179)	(*n* = 18)
Transthoracic echocardiography				
LVEF < 55% or LV GLS > −15%	22 (9.5)	3 (8.8)	16 (8.9)	3 (16.7)
Body plethysmography				
DLCO < 80% of target	51 (22.1)	5 (14.7)	42 (23.5)	7 (38.9)
FVC < 80% of target	23 (10.0)	0 (0.0)	23 (12.8)	0 (0.0)
FEV1 < 80% of target	24 (10.4)	1 (2.9)	22 (12.3)	1 (5.6)
FEV1/FVC < 80% of target	1 (0.4)	0 (0.0)	1 (0.6)	1 (5.6)
6-Minute walk test				
Reduced distance	55 (23.8)	6 (17.6)	43 (24.0)	6 (33.3)
Desaturation during exercise of > 7% or < 90%	26 (11.3	1 (2.9)	21 (11.7)	4 (22.2)
Borg Dyspnea Scale > 6 (at end)	9 (3.9)	1 (2.9)	7 (3.9)	1 (5.6)
Borg Exertion Scale > 6 (at end)	8 (3.5)	0 (0.0)	7 (3.9)	1 (5.6)
Capillary blood gas test				
pO_2_ < 65 mm Hg	10 (4.3)	1 (2.9)	8 (4.5)	1 (5.6)
pCO_2_ > 45 mm Hg	1 (0.4)	1 (2.9)	0 (0.0)	0 (0.0)
Blood tests				
C-reactive protein > 5.0 mg/L	23 (10.0)	1 (2.9)	19 (10.6)	3 (16.7)
Troponin T > 14 ng/L	5 (2.2)	1 (2.9)	3 (1.7)	1 (5.6)
NT-proBNP > 300 pg/mL	3 (1.3)	1 (2.9)	2 (1.1)	0 (0.0)

**Table 3:** Comparison of patients with a clear indication of organ damage due to COVID-19 in Step 2 diagnostics with patients without “functional long COVID”.

Characteristic	Organ Damage Due to COVID-19 (*n* = 16)	Functional Long COVID (*n* = 20)	*p*-Value
Age, mean (SD), year	52.4 ± 15.2	48.0 ± 14.9	0.391 *
Women, *n* (%)	7 (43.8)	12 (60.0)	0.503
Body mass index, mean (SD), kg/m^2^	29.3 ± 4.3	24.5 ± 3.6	0.001 *
COVID-19 history			
Oligosymptomatic/asymptomatic course, *n* (%)	2 (12.5)	3 (15.0)	1
Hospitalization, *n* (%)	3 (18.8)	2 (10.0)	0.637
Invasive ventilation, *n* (%)	2 (12.5)	0 (0.0)	0.19
Therapy with corticosteroids, *n* (%)	3 (18.8)	0 (0.0)	0.078
Therapy with antibiotics, *n* (%)	2 (12.5)	3 (15.0)	1
Other histories			
Cardiac diseases, *n* (%)	1 (6.3)	1 (5.0)	1
Pulmonary diseases, *n* (%)	3 (18.8)	2 (10.0)	0.637
Malignant diseases, *n* (%)	1 (6.3)	0 (0.0)	0.444
Cardiovascular risk profile			
Arterial hypertension, *n* (%)	9 (56.3)	2 (10.0)	0.004
Diabetes mellitus type I, *n* (%)	1 (6.3)	0 (0.0)	0.444
Diabetes mellitus type II, *n* (%)	3 (18.8)	0 (0.0)	0.078
Dyslipidemia, *n* (%)	9 (56.3)	13 (65.0)	0.734
Current/past smoking, *n* (%)	4 (25.0)	5 (25.0)	1

* Student’s *t*-test used.

## Data Availability

The datasets used and/or analyzed during the current study are available from the corresponding author on reasonable request.
